# Provider and patient perceptions of malaria rapid diagnostic test use in Nigeria: a cross-sectional evaluation

**DOI:** 10.1186/s12936-018-2346-x

**Published:** 2018-05-16

**Authors:** Olugbenga A. Mokuolu, Olufemi O. Ajumobi, Godwin N. Ntadom, Olanrewaju T. Adedoyin, Alero A. Roberts, Chimere O. Agomo, Kate U. Edozieh, Henrietta U. Okafor, Robinson D. Wammanda, Friday A. Odey, Ibrahim K. Maikore, Olatayo O. Abikoye, Adekunle D. Alabi, Chiomah Amajoh, Bala M. Audu

**Affiliations:** 1 University of Ilorin, Ilorin, Nigeria; 2 National Malaria Control Programme, Federal Ministry of Health, Abuja, Nigeria; 3 University of Lagos, Lagos, Nigeria; 4 Nigerian Institute of Medical Research, Lagos, Nigeria; 5 New Initiative for the Enhancement of Life and Health (NELAH) Ibadan, Ibadan, Nigeria; 6 University of Nigeria, Nsukka, Nigeria; 7 Ahmadu Bello University, Zaria, Nigeria; 8 University of Calabar, Calabar, Nigeria; 9 Olabisi Onabanjo University, Shagamu, Ogun State Nigeria; 10 University of Jos, Jos, Nigeria; 11 African Field Epidemiology Network, Abuja, Nigeria; 12 National Coordinator National Malaria Elimination Programme, Federal Ministry of Health, Abuja, Nigeria

**Keywords:** Rapid diagnostic test, Malaria, Primary health care workers, Community, Perception

## Abstract

**Background:**

Nigeria commenced a phased programmatic deployment of rapid diagnostic tests (RDT) at the primary health care (PHC) facility levels since 2011. Despite various efforts, the national testing rate for malaria is still very low. The uptake of RDT has been variable. This study was undertaken to determine the provider and patient perceptions to RDT use at the PHC level in Nigeria with their implications for improving uptake and compliance.

**Methods:**

A cross-sectional survey was conducted in 120 randomly selected PHCs across six states, across the six-geopolitical zones of Nigeria in January 2013. Health facility staff interviews were conducted to assess health workers (HW) perception, prescription practices and determinants of RDT use. Patient exit interviews were conducted to assess patient perception of RDT from ten patients/caregivers who met the eligibility criterion and were consecutively selected in each PHC, and to determine HW’s compliance with RDT test results indirectly. Community members, each selected by their ward development committees in each Local Government Area were recruited for focus group discussion on their perceptions to RDT use.

**Results:**

Health workers would use RDT results because of confidence in RDT results (95.4%) and its reduction in irrational use of artemisinin-based combination therapy (ACT) (87.2%). However, in Enugu state, RDT was not used by health workers because of the pervasive notion RDT that results were inaccurate. Among the 1207 exit interviews conducted, 549 (45.5%) had received RDT test. Compliance rate (administering ACT to positive patients and withholding ACT from negative patients) from patient exit interviews was 90.2%. Among caregivers/patients who had RDT done, over 95% knew that RDT tested for malaria, felt it was necessary and liked the test. Age of patients less than 5 years (p = 0.04) and “high” educational status (p = 0.0006) were factors influencing HW’s prescription of ACT to RDT negative patients.

**Conclusion:**

The study demonstrated positive perception to RDT use by HW and among community members with good compliance rate among health workers at the PHC level. This positive perception should be explored in improving the current low level of malaria testing in Nigeria while addressing the influence of age on HW administration of ACT to RDT negative cases.

## Background

Malaria is still a cause of significant morbidity and mortality in Nigeria [1, 2]. The national policy on malaria treatment, which initially encouraged home management and treatment on the basis of clinical diagnosis for all ages, has since the introduction of artemisinin-based combination therapy (ACT) been faced with issues of over-diagnosis and over-treatment with undesirable outcomes [3–7]. Currently, the Nigeria national guidelines recommend a prompt parasitological diagnosis of all febrile suspected malaria cases and administration of recommended ACT to only test positive cases [8]. The mainstay of malaria case management is the identification of the parasite in blood films, which is not always available or feasible at peripheral health facilities in resource-limited settings [9–11]. Conforming to global standards requires committing to parasitological diagnosis prior to initiating treatment with ACT [12].

Rapid diagnostic tests (RDTs) have been found useful as a rapid diagnostic measure in the management of malaria [11, 13]. Their use has reduced the unnecessary administration of anti-malarial medicines and the early diagnosis of other non-malaria illnesses thus reducing treatment cost and morbidity and mortality [12]. Consistent with global best practices and World Health Organization (WHO) recommendations, Nigeria recently updated its guidelines recommending universal testing before treatment [8]. This imposes the need to make diagnostic tools available as peripherally as possible. Deployment of RDTs at primary health centres have been reported elsewhere to be plagued with the ambiguity of policy and abiding mistrust of the efficacy of RDTs to detect malaria resulting in poor compliance to the clinical guidelines for malaria case management following negative RDT results [13–15].

In the Nigerian context, while there has been the deployment of RDT to peripheral centres, a robust monitoring mechanism has been lacking. This has made it difficult to accurately ascertain the extent of the distribution of RDTs, utilisation by health workers, the acceptance by patients, the challenges its introduction has posed to treatment of RDT negative patients [16], competence of the health workers in its use [17], knowledge, perception and attitude of health workers to it [18], and the influence on the use of ACT since it was introduced. Without the knowledge of the, it would be difficult to identify gaps to be filled and challenges to implementation. The current effort is the first attempt to carry out a countrywide sectoral analysis of RDT in Nigeria. A similar study in the private sector of Nigeria health system has been published [19] and the public sector aspect needs to be addressed. In view of these gaps which include limited data on the utilization of RDT results in Nigerian public health sector to influence treatment and management options in RDT negative febrile illness, the National Malaria Elimination Programme evaluated provider and patient perceptions on the use of RDT in the malaria case management at primary healthcare level in Nigeria.

## Methods

### Study areas

**Fig. 1:**
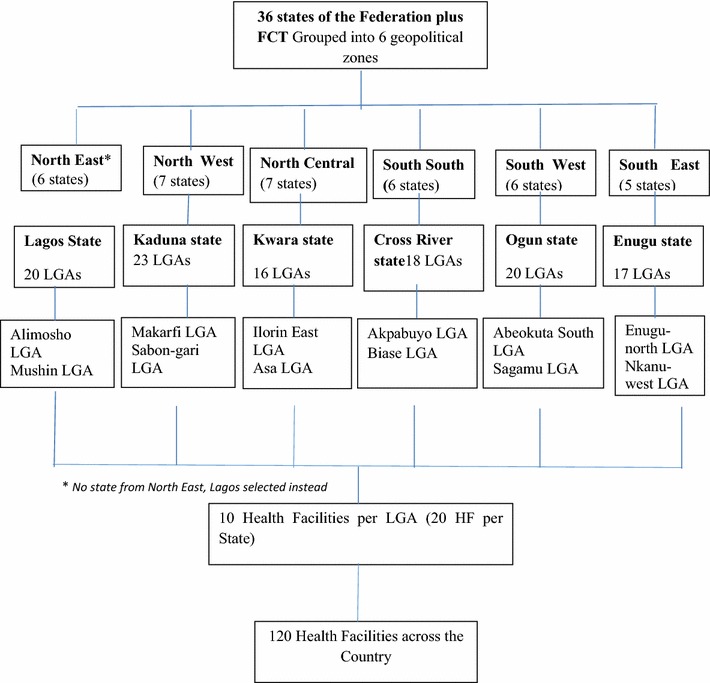
Description of Sampling Strategy

Nigeria has various ecological zones with vegetation changing from Sahel savannah in the far north followed by Sudan savannah merging into guinea savannah in the middle belt, then rain forest in the south and mangrove forest in the coastal areas. Hitherto, malaria transmission and risk had been judged to be high across the country [20]. However, latest information has provided evidence of a progress divergence of in-country variation in malaria endemicity [21]. Nigeria has a population of about 160 million with 40% access to public health facilities [20]. The country has 36 states and a Federal capital territory across 6-geopolitical zones (Fig. 1). Each state is composed of local government areas as the lowest tier of governance. There are approximately 10 primary health care centres (PHCs) per LGA. *Plasmodium falciparum* is the predominant species in about 94% of diagnosed malaria cases [22].

This study was conducted in 120 primary health care facilities located in 12 local government areas (LGAs) across six states from the geopolitical zones of Nigeria. Six states viz; Cross River, Enugu, Kaduna, Kwara, Ogun were randomly selected, one from each of geopolitical zone. Kwara and Kaduna represented north-central and north-west zones in northern Nigeria; while Cross River, Enugu, Lagos, Ogun represented south–south, south-east and south-west zones in the south. The malaria prevalence was 26.1, 10.5, 36.7, 26.4 and 14.7% in Cross River, Enugu, Kaduna, Kwara and Ogun states, respectively, but nil in Lagos [22]. The study was conducted in January 2013.

### Study design

This was a cross-sectional multi-stage random sampling survey.

#### Sampling technique

Multi-stage random sampling technique was used. Nigeria has 774 LGAs distributed across 36 states and one Federal Capital Territory, within six geo-political zones. In the first stage, 1 state per geopolitical zone was selected. States in the north-east geopolitical zone were excluded because of security reasons, while Lagos state was selected as a result of coastal location and status as emerging mega city status. In the second stage, two non-contiguous LGAs were randomly selected per state. Ten primary healthcare clinics (PHCs) per LGA were then included in the study (Fig. 1). The selected PHCs had trained staff on malaria case management training and RDT use, recording and reporting tools, and managing a consistent supply of RDTs and ACT medicines. The RDTs were histidine-rich protein based. However, brand specifications were based on existing programmatic arrangement for the supply of selected states. Standard Diagnostic *Bioline* in Enugu, *Carestart* and *First Response* in Kwara and *First Response* in all other states.

#### Data collection

**Fig. 2:**
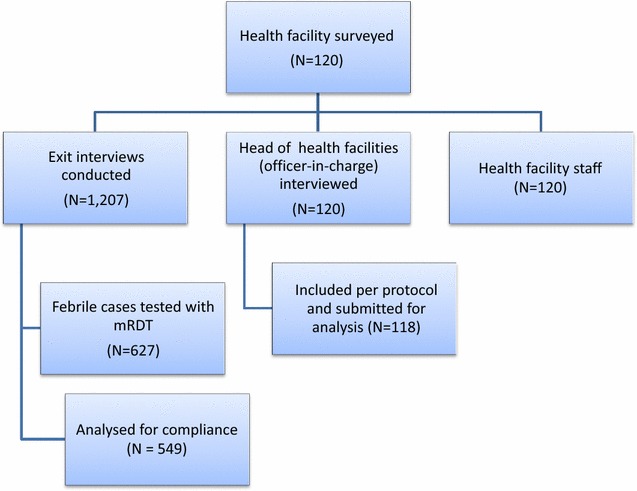
Data Management Algorithm for data collection and recruitment of subjects

Study instruments were pre-tested at other selected sites excluded from the study, to standardize the tools and ensure consistency of source documents. Eighty-four data collectors were recruited and trained. Each state team comprised of a state team leader, 12 data collectors and a transcriber. Structured questionnaires were administered to each health worker administering treatment to assess knowledge and perception of health worker on RDT use, operational issues with RDT use, and health workers’ treatment practices with regards to RDT results. Another questionnaire was administered to the officer-in-charge (head) of the health facility to assess clinic workflow in relation to conduct of RDTs (Fig. 2).

Patient exit interviews were conducted to assess patient perception of RDT from ten patients/caregivers who met the eligibility criterion and were consecutively selected in each PHC and interviewed after their visit, 120 PHCs in all. Eligibility criterion was a patient who had presented with a febrile illness or a caregiver whose child had presented with a febrile illness of 24 h duration and gave consent to participate in the study. An additional seven interviews were conducted in a health facility. Overall, 1207 exit interviews were conducted (Fig. 2).

Community members, each selected by their ward development committees^1^ in each LGA were recruited for focus group discussion (FGDs). Male and female FGD sessions were conducted separately. Twenty-four community-based FGD sessions were conducted in local languages, two in each LGA. They captured information on the prevalence of malaria and other febrile conditions in the community, fever management practices; compliance by health workers to testing before treatment, awareness about RDT, acceptability, and willingness for the continued use of RDT.

### Data processing and analysis

Data collation, validation, and initial cleaning were done on the field across the states.

#### Quantitative

Responses on the questionnaires and checklists were cleaned and entered in Epi-info version 3.5.3./SPSS version 16. Merging of the national data and central analysis was carried out by a team of the study biostatisticians. Variables were presented as proportions in frequency tables and charts after data cleaning. Bivariate analysis was conducted to elicit factors associated with the programmatic deployment of RDTs. Chi square test was used to compare categorical variables. Results were considered significant at α < 0.05.

### Qualitative data

Focus group discussion notes and audio tapes were transcribed and translated from local language into English. Similar themes were identified and categorized. Content analysis and descriptive interpretation of data was done.

### Compliance with RDT results

#### Definition of compliance indicator

For this study, compliance was defined as the (Number of patients with RDT positive results who received ACT) + (Number of patients with RDT negative results who did not receive ACT)/total number of patients on whom RDT test for malaria was performed × 100. This was calculated from self-reporting of results of RDT test and prescription or not of the ACT based on the test result, during patient exit interviews.

## Results

### Health workers knowledge and perception of RDTs

The 120 health workers interviewed had all been previously trained on malaria case management. Of these, 78.8% knew what the acronym ‘RDT’ meant, 99.2% knew that the test diagnosed malaria and that the specimen tested was blood. One hundred and eight (90%) correctly reported the quantity needed as a drop and 99.2% admitted knowing how to do an RDT. Time taken to do the RDT was correctly reported as 15–20 min by 60.9% of health workers and 90.8% said they would use the test results to determine who should get ACT.

Health workers reported unanimously that they would use the RDT results to determine whether ACT medicines were prescribed. Reasons given were that they have confidence in the results (95.4%), it would reduce the unnecessary use of ACT (87.2%), permit the consideration of other conditions (67.9%) and because it gave them more confidence than microscopy results (43.1%).

### Acceptance of RDT

Most of the 120 health workers, 112 (93.3%) said RDT is not difficult to carry out, 114 (95.0%) said that RDT was not rejected by patients, 107 (89.2%) said RDT is not time-consuming and 108 (90.0%) reported that it did not interfere with clinic activities.

### Health workers practices in relation to RDT results

**Fig. 3:**
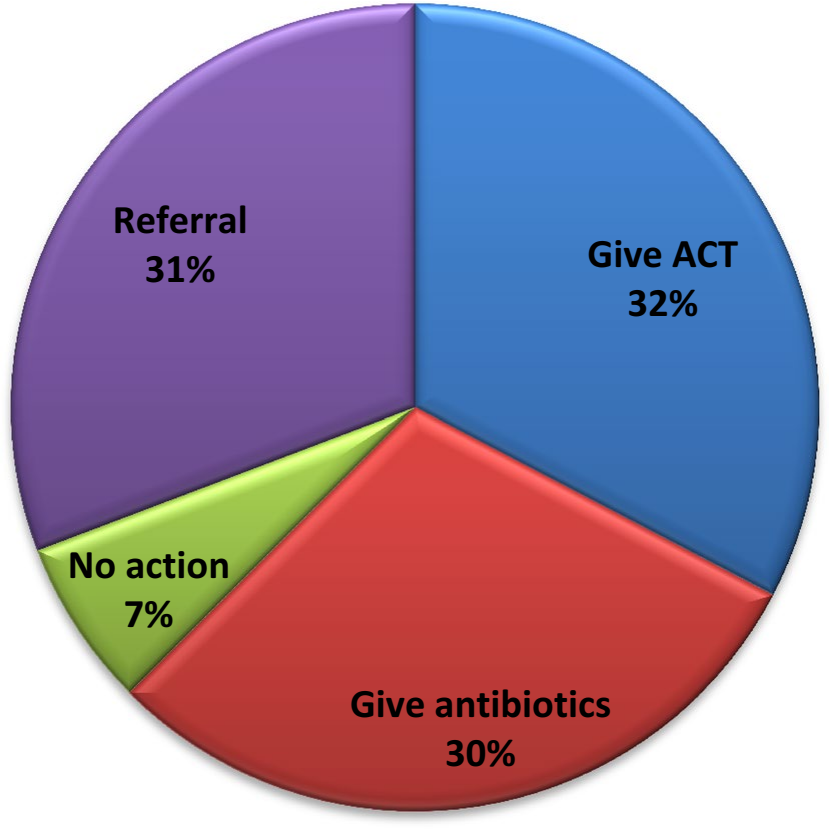
Health workers reported actions taken when RDT is negative

88 (73.3%) health workers, reported that they comply with RDT results. However, if the test was negative 104 (86.7%) reported their actions; 32% of the health workers reported they would prescribe ACT, 30% would administer antibiotics, 31% would refer such patients, and 7% would take no further action (Fig. 3).

### Clinic workflow and conduct of RDTs

Testing with RDT took place at patient registration in 11.9% of cases and during consultation in 44.9% of cases (Table 1). When RDT was done during the consultation process, 77.8% said they would continue consultation with another patient while waiting for the results. In 56.7% of cases when RDT is done after consultation, 97.1% said the patient comes back with the results.

**Table 1:** Malaria testing and workflow (reported by officers’-in-charge)

State	When was RDT carried out n (%)			Total n
	After consultation	Before consultation	During consultation	
Cross River	6 (31.6)	2 (10.5)	11 (57.9)	19
Enugu	7 (35)	3 (15)	10 (50)	20
Kaduna	9 (45)	0 (0)	11 (55)	20
Kwara	11 (55)	6 (30)	3 (15)	20
Lagos	15 (75)	1 (5)	4 (20)	20
Ogun	3 (15.8)	2 10.5)	14 (73.7)	19
Total	51 (43.2)	14 (11.9)	53 (44.9)	118

### Effect of RDT results on health workers’ prescription practices

#### Compliance of health workers to RDT results from patients exit interviews

Among the 1207 exit interviews conducted, 549 (45.5%) had received RDT test. Of the tested 549 patients, 292 (53.2%) received ACT for positive RDT results and 203 (37.0%) did not receive ACT for negative RDT results. The overall compliance rate from patient exit interviews was 90.2% (Table 2).

**Table 2:** Compliance with RDT results, patient exit interviews, primary health care centres in Nigeria—2012

State	Exit interviews	rdt use	RDT pos with ACT	RDT neg without ACT	% compliance
		a	b	c	$\frac{b + c}{a} \times 100$
C/River	200	116	88	13	87.1%
Enugu	205	–	–	–	–
Kaduna	200	198	41	144	93.4%
Kwara	200	156	114	35	95.5%
Lagos	202	61	41	10	83.6%
Ogun	200	18	8	1	50.0%
Total	1207	549	292	203	90.2%

#### Patient and caregiver factors influencing the use of RDT results

The results of the bivariate analysis of some factors tested against their possible influence on the prescription of ACT for RDT negative cases showed that age of the patient less than five and “high” educational status was significantly associated with prescribing ACT for RDT negative cases. The symptom complex at presentation however did not influence the use of ACT in RDT negative situations (Table 3). From the qualitative data, it was discovered that the people who were more educated demanded the prescription of ACT as a matter of right even when RDT results were negative; they claim that *“government has already made it available and so they should be given”.* In Enugu state, RDT was not being used for the period under review because of a general belief that it was not sensitive.

**Table 3:** Factors associated with administration of ACT to RDT negative patients on exit interviews of caregivers of children below 5 years

Age (years)	RDT neg with ACT		TOTAL	χ2	*p* value
	No	Yes			
Age					
< 5	59 (72.8)	22 (27.2)	254	4.187	0.04
≥ 5	145 (83.8)	28 (16.2)			
Education					
None/incomplete secondary school	162 (85.3)	28 (14.7)	250	11.83	0.0006
≥ complete secondary school	39 (65.0)	21 (35.0)			
Symptoms at presentation					
Fever and other signs/symptoms	154 (79.8)	39 (20.2)	253	0.101	0.75 (NS)
Fever alone	49 (81.7)	11 (18.3)			

*NS* No significant difference

### Patients’/caregivers’ opinions of RDT use

The respondents who had RDT done knew that RDT tested malaria (96.7%), felt it was necessary (94.9%) and liked the test (98.8%) because it allowed them to be sure of what was wrong (92.3%).

## Discussion

This study is the largest and most representative on RDT deployment in Nigeria; providing some significant data related to current practices. The findings are expected to prove useful in other programmatic activities, especially monitoring and evaluation and in the quantification of anti-malarial commodities.

From this study, fever prevalence was 52% among outpatient attendees. The use of RDT was 38.8% at the PHC level with malaria positivity rate by RDT of 59.1%. These are important baseline figures against which future changes can be measured. It is, however, possible that the denominator with respect to the total number of febrile subjects may have been larger than that reported, thus giving a slightly exaggerated malaria prevalence. This is because, in some of the interviews, the health workers admitted that they only entered the data of those that test positive to RDT results. Such misconceptions will, therefore, have to be corrected as the country scales up RDT implementation, in order to better reflect the true prevalence of malaria at the health facility levels. While taking into consideration the methodological differences between the National Malaria Indicator Survey and the current study, the malaria prevalence of about 60% in this study was comparable with the 45% RDT positivity in the 2015 MIS [22].

This study also showed various outcomes in relation to the impact of RDT result on prescription practices using the report of patient exit interviews as a proxy. The compliance rate of 90.2% for a newly implemented programme is significant. This is consistent with observations from other countries that have shown high penetration of RDTs among workers at the PHC levels [23, 24]. About one-third would give antibiotics, one-third would refer and one-third would give ACT. This is similar to reports from Tanzania [25]. There has been a persistent challenge regarding the best actions in the presence of RDT negative febrile patients. It is unlikely that this may be easily resolved by training and other communications. This is largely because a single recommendation that would capture the variables involved in the management of RDT negative febrile cases is a challenge at the moment. However, in the short term, there must be a sustained effort at ensuring compliance with RDT results, through periodic capacity development, supervisory visits and use of health workers forum to reinforce best practices. Ultimately having an integrated rapid diagnostic test kit that would simultaneously test for malaria, bacterial infections and possible viral pathology may be the direction to explore [16, 26, 27].

Sub-group analysis with complementary tools further indicates that the age of the subject, parents level of education also play significant roles in making health workers prescribe ACT to RDT negative subjects. This is probably a novel insight to other challenges influencing ACT use in RDT negative subjects. It is an “adverse effect of governments’ good intention” in making ACT medicines freely available. The implication of this is that while addressing the health workers, in order to strengthen their compliance, the contributions of the public or community members to health workers’ compliance with RDT results cannot be ignored. This means that information, education, and communication/behavioural change communication (IEC/BCC) strategies should include extra measures to target members of the community with a minimum of completed secondary education.

There was an irregular supply of the RDT kits in some health facilities and in others, incidences of stock out were quite frequent thereby interfering with the process of creating a practice culture in the management of febrile illness at the peripheral level. At the time this study was undertaken some of the health workers clearly complained of stock out and reiterated that it could take weeks before their supply are replenished. This obviously does not help the development of a programme rather it gives rise to frustration and apathy. It is evident that the procurement and supply management chain will need to be strengthened.

It is speculated that there may have been probable doubt on the part of the health workers on the outcome of the RDT testing when compared with the clinical picture. In the event of doubt about the outcome of the RDT testing, a second test which may be another RDT with a different mechanism of action or a microscopic examination is carried out. If both results are negative, then the cause of the fever cannot be malaria [28]. In addition, improving quality assurance system for RDTs and the use of positive control wells on the field will boost providers’ confidence in results of RDTs [29].

Health workers should practice evidence-based medicine and avoid empirical treatment. This would conserve resources and reduce the pressure on ACT hence preventing early resistance to the drug [30]. In spite of all these, the overall compliance of 90.2% was obtained. This is comparable to 88 (74.6%) risk of health workers who reported during the interview that they comply with RDT results. It can be improved upon until it becomes a standard of care.

This study is descriptive, thus the influence of parental level of education on prescription of ACTs to RDT negative subjects will require further investigation.

## Conclusions

This study provides experience with RDT implementation in a large and diverse country like Nigeria. It demonstrated positive perception to RDT use by HW and among community members with good compliance rate among HW at the PHC levels. There is need to explore this positive perception in improving the current low level of malaria testing in Nigeria through strengthening of distribution systems and other operational constraints. Despite these challenges that may be associated with scale-up efforts for RDT use, its implementation at PHC level in Nigeria is feasible, and acceptable with a significant compliance rate to RDT results; 90% by any standard is an acceptable result.

## Footnotes

1 Ward development committee is a voluntary body established to promote the ward as a basic unit of local governance and as a “planning unit” for local community development. It comprises a councillor, no more than ten other people with at least three of whom must be women, resident in the ward and elected by all residents at a public meeting. UNDP Niger Delta Pilot Local Development Programme—Operational Manual, August 2007.
